# Laparoscopic Approach to Severe Liver Injury in a Patient With Blunt Abdominal Trauma

**DOI:** 10.7759/cureus.36568

**Published:** 2023-03-23

**Authors:** Hilmi Yazici, Orkhan Verdiyev

**Affiliations:** 1 General Surgery, Marmara Üniversitesi Pendik Eğitim ve Araştırma Hastanesi, İstanbul, TUR; 2 General Surgery, Marmara Üniversitesi Pendik Eğitim ve Araştırma Hastanesi, Istanbul, TUR

**Keywords:** liver trauma, laparascopic surgery, laparoscopy, liver, blunt trauma

## Abstract

With laparoscopic surgery becoming more popular in surgical practice, laparoscopic approaches in trauma patients have increased. Non-operative management is the standard treatment algorithm in patients with blunt abdominal trauma who are hemodynamically stable and have sustained a liver injury. However, laparoscopy is a safe and feasible method for exploration, irrigation, and treatment if a surgical intervention is needed in this group. In this study, we aimed to present a case of liver injury in blunt abdominal trauma and its management laparoscopically.

A 22-year-old male was admitted to the Marmara University Hospital’s Emergency Unit of a tertiary center following a truck accident. The patient was hemodynamically stable at admission. CT scan showed a grade IV liver laceration with hemoperitoneum. The patient was transferred to the observation room. After three hours, the patient’s hemoglobin value decreased from 14.6 g/dl to 8.4 g/dl, and the mean arterial blood pressure decreased to 60 mmHg. The patient’s heart rate increased to 125, and peritonitis was evident on the abdominal examination. Emergent laparoscopy was performed on the patient. Grade IV liver laceration with no active bleeding was observed. After peritoneal irrigation, surgery was terminated. With the developments in minimally invasive procedures, laparoscopic approaches were used more frequently in trauma patients. In the referral and experienced centers, laparoscopy could be an appropriate way to avoid unnecessary laparotomies.

## Introduction

As a result of improvements in minimally invasive surgical techniques, laparoscopic surgery has become more critical in every part of surgical practice. It was well described in liver surgeries as a safe and feasible approach [1]. Laparoscopic surgery for penetrating abdominal traumas was well described before; however, it was unclear for blunt abdominal traumas (BAT). In BAT, liver injuries (LI) were seen in 38 % of the patients, and only 7.5% had grade IV-V injuries [2]. According to the latest guidelines, almost all hemodynamically stable patients with LI should be managed with non-operative management (NOM) [3]. However, immediate surgical interventions should be performed in hemodynamically unstable patients at admission or those who were becoming unstabilized during the observation period. This study aims to present a case report that needed emergent surgery and was managed with a laparoscopic procedure.

This article was previously presented as a meeting abstract at International Capital Conference Multidisciplinary Scientific Research on 13-14 July 2022.

## Case presentation

**Figure 1:**
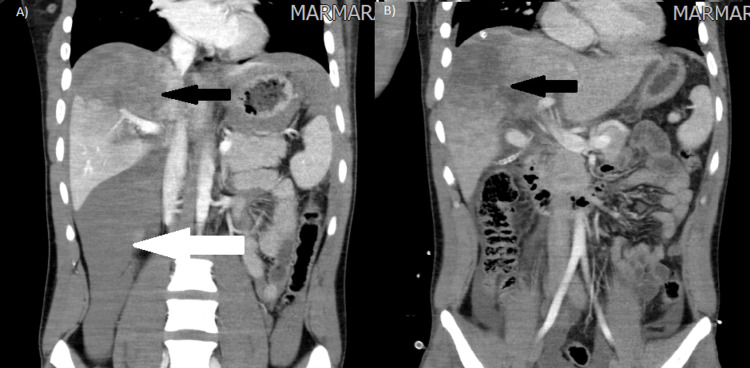
(A) CT findings at admission ( The black arrow shows the laceration area on the liver, and the white arrow shows the massive intraperitoneal hemorrhage; (B) CT findings after the operation (The black arrow shows the laceration area postoperatively).

**Figure 2:**
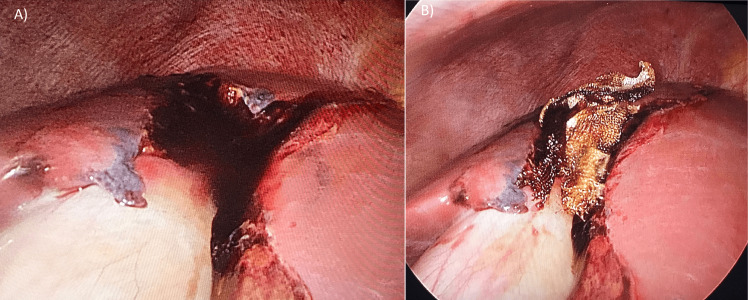
(A) Liver Injury among Segments 4-5-8 (B) Injury line after hemostasis

A 22-year-old male patient was admitted to Marmara University Hospital’s Emergency Department with blunt abdominal trauma after a truck accident. The patient was hemodynamically stable with 122/75 mmHg arterial blood pressure and 81 heart rate at admission. During the physical examination, there was only pain in the right upper quadrant of the abdomen. In the first laboratory results, the hemoglobin value was 14.6 g/dl, and other laboratory findings were also normal. Computerized Tomography (CT) was performed, and it showed a grade IV liver injury, according to the American Association for the Surgery of Trauma (AAST) (3) (Figure 1A). The patient was transferred to the observation room in the general surgery department. A controlled physical examination and laboratory test were performed three hours after admission. Arterial blood pressure was decreased to 90/62 mmHg, and heart rate was increased to 125. Because of the findings of peritonitis and a decrease in hemoglobin level to 8.4 g/dl, it was decided to perform a surgical intervention. Two 5 mm trocars and one 10 mm trocar were placed for laparoscopic exploration. A grade IV laceration extending along Segments 4 and 5-8 of the liver was detected (Figure 2A).

Nevertheless, there was no active bleeding on the injury site. After total abdominal exploration, there was no additional injury in the intraperitoneal cavity, and peritoneal irrigation was performed. After that, Surgicel Absorbable HemostatTM (Ethicon Endo-Surgery, Cincinnati, OH, USA) was placed upon the injury area (Figure 2B), and after the drain replacement, the operation was terminated. There was no fluid collection in control CT on the fourth postoperative day (Figure 1B). Enteral nutrition was started on the first day postoperatively, and on the sixth day after surgery, the patient was discharged from the hospital. 

## Discussion

Although the patient was hemodynamically stable in the observation, complications like biliary leaks (BL) or bleeding might occur, especially in high-grade liver injuries. BL was seen at 0.5 % to 21 % in complicated LI [4]. In recent years, minimally invasive methods were used for these more often to manage complications after LI. Ultrasound-guided biliary intraperitoneal drainage combined with Endoscopic Retrograde Colangiopancreotoghrapy (ERCP) effectively treats biliary leakage after severe LI [4,5]. Therefore, intra-arterial embolizations are also used for delayed liver bleedings during performing NOM [6]. However, surgical interventions should be performed without peritonitis or unstable hemodynamic parameters. In this case, It was thought that the peritonitis might be caused by hemoperitoneum, and emergent laparoscopy was performed because of findings of peritonitis and unstabilized physical parameters. Although the role of laparoscopy remains unclear in BAT, some studies showed that laparoscopic approaches could be a safe and effective method [7,8]. It has various advantages, such as reduced wound healing time, reduced postoperative adhesions, and faster recovery [9]. It provides a direct vision to the exploration and irrigation of the peritoneum to the surgeon, so unnecessary laparotomies are avoided. Furthermore, randomized trials are needed to evaluate standard surgery methods for BAT with severe LI.

## Conclusions

Laparoscopic surgery can safely be performed on trauma patients, which might prevent some patients from unnecessary laparotomies. The majority of BAT patients can be followed without surgery with close observation. Once the patient shows symptoms of peritonitis or becomes vitally unstabilized, laparoscopic exploration could be a choice for clinicians. However, the role of laparoscopy in blunt traumas is still controversial, and further studies are needed on this topic.
